# Lipomas of the oral cavity

**DOI:** 10.1016/S1808-8694(15)31182-4

**Published:** 2015-10-19

**Authors:** Belmiro Cavalcanti Do Egito Vasconcelos, Gabriela Granja Porto, Suzana Célia De Aguiar Soares Carneiro, Ruth Lopes De Freitas Xavier

**Affiliations:** 1 PhD. Postgraduate Program Coordinator - UPE; 2 DDS. Specialist in bucco-maxillo-facial surgery and traumatology - Dentistry School of Pernambuco. M.S. Student in bucco-maxillo-facial surgery and traumatology - Dentistry School of Pernambuco; 3 DDS. Specialist in bucco-maxillo-facial surgery and traumatology - Dentistry School of Pernambuco. M.S. Student in bucco-maxillo-facial surgery and traumatology - Dentistry School of Pernambuco; 4 DDS. Specialist in bucco-maxillo-facial surgery and traumatology - Dentistry School of Pernambuco. M.S. Student in bucco-maxillo-facial surgery and traumatology - Dentistry School of Pernambuco

**Keywords:** tongue, mouth mucosa, oral, pathology

## INTRODUCTION

Intra-oral lipomas are benign and relatively rare tumors, although they occur with higher frequencies in other areas, most especially the back, abdomen and shoulders of adults^1^, ^2^, ^3^. This paper describes three cases of lipoma and their symptoms, histological characteristics and anatomical findings are discussed as well.

## CASES REPORT

### Case 1

**Figure 1:**
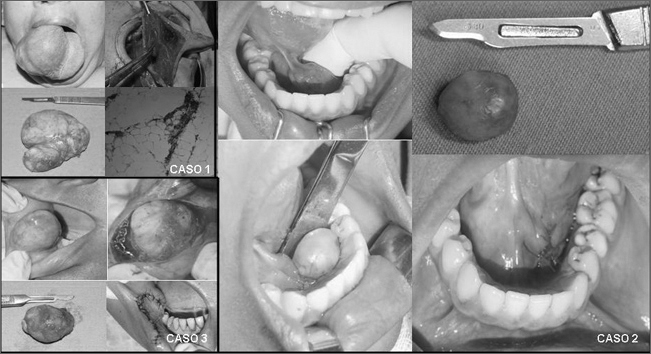
Oral cavity lipomas: (from left to right). Case 1: Preoperative, transoperative aspect, macroscopy and histology. Case 2: preoperative, macroscopic aspect of the lesion, transoperative and immediate postoperative aspects. Case 3: Preoperative, transoperative aspect, macroscopy and immediate postoperative aspect.

Female, 60 year old, with 3 years of accelerated development which caused a rare large size of this type of lesion. Treatment of choice was the surgical removal of this lesion after incisional biopsy of the tumor and diagnostic confirmation of lipoma. The patient is currently under observation after 6 months of follow up, without complications (Figure 1).

### Cases 2 and 3

Cases 2 and 3 are related to smaller lipomas, most commonly found in the oral cavity. In both cases, the patients were females, with ages ranging between 30 and 45 years, respectively. In case 2, the lesion was in the inferior lingual region; and in case 3, it was in the jugal mucosa (Figure 1). Both were treated by surgical excision of the whole lesion and the material was referred to histopathology, with later confirmation of lipoma.

## DISCUSSION

Lipomas are histologically very similar to normal fat tissue1. However, its metabolism is quite different from that of the normal tissue, because its lipids are not available for our normal metabolism^1^. Patients are usually symptom-free, and the lesion is usually a yellowish submucosal mass attached by a sessile base or pedicle^5^.

It bears variable sizes, from small −10mm masses, to large fat lesions and happen most frequently in the oral mucosa. Tongue, mouth floor, jugal mucosa, vestibule, palate, lips and gums are the most common sites, in descending order^5^. It more frequently affects persons with more than 40 years of age and bears equal intra-oral gender distribution^4^.

Although trauma, infection and other factors have been proposed as etiological agents for lipomas, their etiology remains unknown. The treatment of choice is conservative surgical excision and recurrence is rare, as is its malignant transformation^4^,^5^.
